# Evaluation of a Computed Tomography-based Technique for Predicting Atrial Fibrillation Recurrence Following Ablation Using an Adjusted Skeletal Muscle Index

**DOI:** 10.31083/RCM26933

**Published:** 2025-04-23

**Authors:** Pingchuan Ma, Zhicheng Gao, Jiaqi Bao, Yilan Hu, Pengfei Sun, Qiqi Yan, Lifang Ye, Lihong Wang

**Affiliations:** ^1^The Second Clinical Medical College, Zhejiang Chinese Medical University, 310000 Hangzhou, Zhejiang, China; ^2^Heart Center, Department of Cardiovascular Medicine, Zhejiang Provincial People’s Hospital (Affiliated People’s Hospital, Hangzhou Medical College), 310000 Hangzhou, Zhejiang, China

**Keywords:** atrial fibrillation, skeletal muscle index, recurrence, computed tomography, sarcopenia

## Abstract

**Background:**

This research focuses on the unresolved question of how low muscle mass influences the likelihood of atrial fibrillation (AF) recurrence after ablation treatment. Despite the growing body of evidence highlighting the importance of muscle mass in cardiovascular health, the specific impact of low muscle mass on the recurrence of AF following ablation has yet to be well-established. Thus, this study evaluated the relationship between a low computed tomography (CT)-based skeletal muscle index (SMI) of muscle sites at the fourth thoracic level (T4-SMI) and AF recurrence post-radiofrequency ablation. Furthermore, this study aimed to determine whether the T4-SMI is a predictive marker for AF recurrence.

**Methods:**

This study included 641 patients with AF who underwent radiofrequency ablation. T4 muscle sites were determined using SliceOmatic software. Height- and body mass index (BMI)-corrected SMIs were calculated.

**Results:**

The lowest quartile in the T4-SMI group was defined for each sex as the “low SMI” group. The height-adjusted T4-SMI thresholds were 69.7 cm^2^/m^2^ for males and 55.91 cm^2^/m^2^ for females. The BMI-adjusted thresholds were 8.10 cm^2^/kg/m^2^ for males and 5.78 cm^2^/kg/m^2^ for females. After potential confounder adjustment, low T4-SMI was associated with a higher risk of AF recurrence. The correlation between T4-SMI (height) and AF recurrence was fully validated by constructing multiple models, and adjusting for different covariates barely altered the results. Fully adjusted models suggested that compared with the fourth T4-SMI (height) quartile, the risk odds ratio (OR) with a 95% confidence interval (CI) of the “low SMI” group was 1.57 (0.76–3.22). Finally, subgroup analysis and interaction according to gender, age, overweight/obesity, hypertension, or diabetes indicate that the differences between different layers are not significant.

**Conclusions:**

Low CT-based BMI- or height-adjusted T4-SMIs were risk factors for AF recurrence post-radiofrequency ablation. A lower T4-SMI (height) significantly correlated with AF recurrence post-ablation, regardless of gender, age, or overweight/obesity. The height adjustment performed better than the BMI adjustment in that regard.

## 1. Introduction

The prevalence of atrial fibrillation (AF) in the Chinese adult population has been reported to be 1.6%, which increases with age [1]. Catheter ablation is considered an alternative treatment option to pharmacotherapy with antiarrhythmic drugs due to its superior ability to maintain sinus rhythm. Although pulmonary vein isolation can successfully resolve AF in most cases, recurrence is possible and is dependent on factors, such as patient’s age, the type and duration of AF, atrial function, and the presence of comorbid metabolic diseases. Obesity, defined as a severely elevated body mass index (BMI), has been shown to increase AF risk [2]. However, more recent studies have demonstrated that the lean body mass, as opposed to obesity-specific parameters, is the main anthropometric risk factor for the AF development [3, 4]. A Danish longitudinal study reported that greater lean body mass, estimated via bioelectrical impedance analysis (BIA), was associated with an increased risk of AF [4]. Sarcopenia, as defined by the 2019 criteria by the Asian Working Group for Sarcopenia (AWGS), is associated with an elevated risk of cardiovascular disease (CVD) in middle-aged and older Chinese adults [5]. In middle-aged and older adults without clinical heart failure, the presence of sarcopenia, assessed using Dual-Energy X-ray Absorptiometry (DXA), has been shown to be significantly associated with the occurrence of AF [6]. However, its relationship with clinical outcomes after radiofrequency ablation in the general population remains unknown. Assessment of the cross-sectional area of skeletal muscle using individual cross-sectional computed tomography (CT) scans is commonly used as a valid proxy for whole-body muscle mass and to determine the skeletal muscle index (SMI), which can be adjusted in various ways [7]. CT is simpler to perform and more effective compared with DXA and BIA in assessing the presence of sarcopenia. Additionally, CT is unaffected by the presence of excess fat or bodily fluids [8]. Therefore, the imaging modality can be used to assess preoperative “low SMI” to measure skeletal muscle area (SMA) at the fourth thoracic (T4) level (T4-SMA) [9]. The present aimed study to investigate the relationship between preoperative CT-based SMI values derived from height- or BMI-adjusted SMA measurements at the T4 level (T4-SMI) and the likelihood of AF recurrence following radiofrequency ablation. We hypothesized that at the T4 level, low SMI obtained by quantifying chest skeletal muscles on single-layer axial chest CT may be a strong risk factor for recurrence following radiofrequency ablation in AF patients.

## 2. Materials and Methods

The data collection work for this study began in May 2023 and ended in November 2023. The data analysis was conducted in December 2023, and the final report was finalized in March 2024.

### 2.1 Study Population

**Fig. 1.**
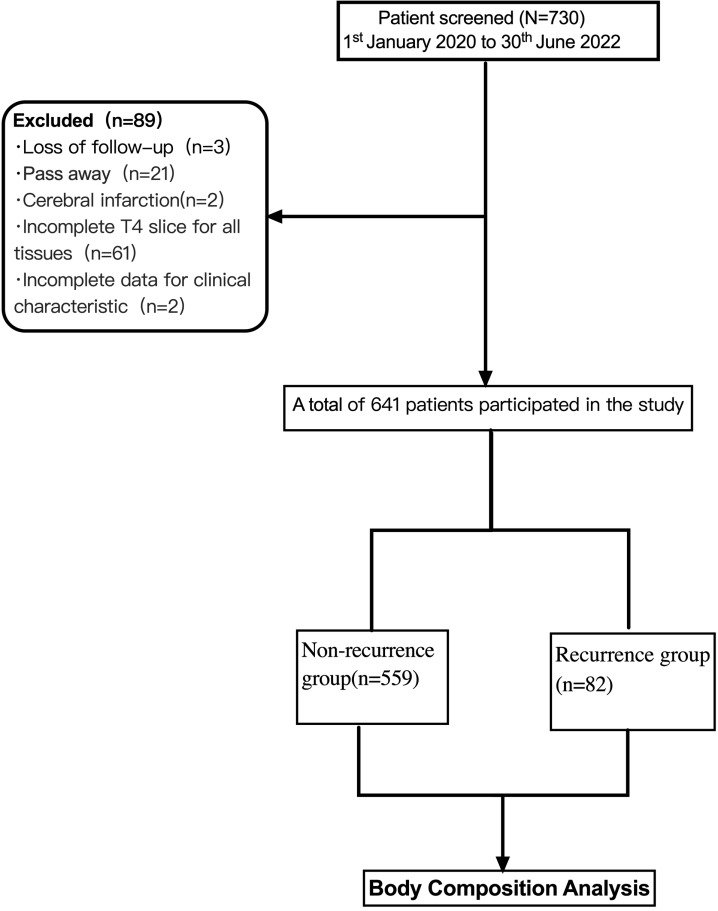
**Flow chart of the study**.

Patients with non-valvular, drug-refractory AF who underwent catheter ablation from January 2020 to June 2022 at Zhejiang Provincial People’s Hospital were eligible for inclusion in the present study. The flow diagram depicting the selection process of subjects in this study is shown in Fig. 1. The exclusion criteria were: patients with treatable causes of AF (hyperthyroidism), rheumatic heart disease, congenital heart disease, autoimmune diseases, severe liver and renal dysfunction, and malignant tumours, and those who did not undergo chest CT examination. Of 730 cases, 3 were lost to follow-up, 21 died (from advanced age, new coronavirus infections, or other factors), 2 experienced cerebral infarcts, 61 had no CT imaging data, and 2 lacked information on clinical characteristics. Eventually, 641 (401 male and 240 female patients) were included in the study.

### 2.2 Patient Management, Data Collection, and Clinical Follow-up

All participants in this study underwent successful radiofrequency ablation of AF. After the surgery, all patients need to take warfarin or new oral anticoagulants orally for 2–3 months to ensure anticoagulant efficacy and prevent the risk of thrombosis. For patients with frequent episodes of preoperative fibrillation, they are advised to take amiodarone, propafenone or beta-blockers for 2–3 months after surgery to control symptoms and reduce recurrence, with amiodarone being the most commonly used. In order to avoid gastrointestinal adverse reactions during medication, all patients must take proton pump inhibitors, gastric mucosal protectants, and prokinetic drugs orally for 1–2 months after discharge. Beta-blockers such as Metoprolol Succinate Sustained-release Tablets, Metoprolol Tartrate Tablets are usually half a pill/one to start with, and the dosage is adjusted later according to his blood pressure and heart rate. The actual dosage of medication should be determined based on the patient’s specific condition and the doctor’s guidance. Baseline information were collected from patient admission records. After discharge, regular outpatient follow-up visits were planned at the end of the first month and every 3–6 months thereafter; they included routine surface electrocardiography and 24-h ambulatory electrocardiography to assess AF recurrence. Patients were recommended to have the additional examinations when they had suspicious symptoms that related to arrhythmias. Follow-up visits were conducted by telephone for out-of-towners and those with limited access to transportation. AF recurrence was defined as the presence of AF, atrial flutter, or tachyarrhythmia ≥30 s in duration 3 months after catheter ablation (with a blanking period of 3 months). An atrial arrhythmia lasting longer than 30 s that had occurred within the previous 3 months was classified as an early recurrence.

### 2.3 Definition of Low SMI and SMI Assessment Methods

**Fig. 2.**
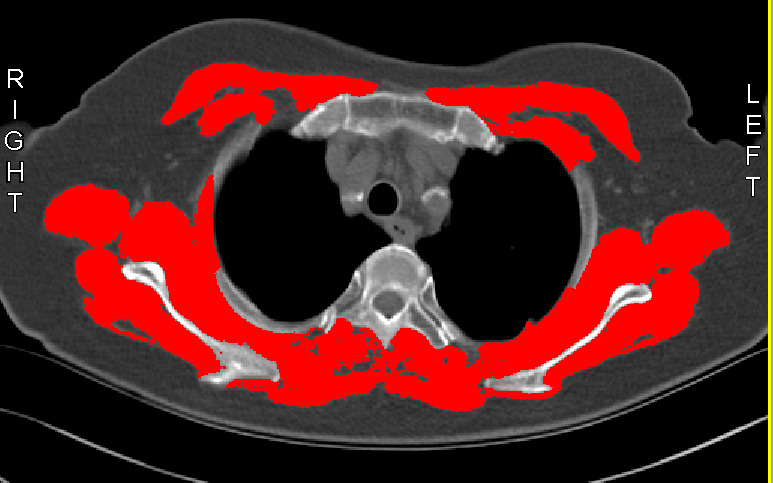
**Example of a fourth thoracic (T4) computed tomography scan with tissue quantification, with skeletal muscle settings shown in red in the SliceOmatic software (version 5.0; Tomovision, Montreal, QC, Canada)**.

BMI was determined from dividing weight by height squared (kg/m^2^). In the present study, preoperative chest CT images were used to retrospectively quantify the SMA of the chest muscles from T4-level imaging, using hounsfield units (HU) thresholds and representations to differentiate between tissue types: CT-measured HUs ranging between –29 and 150 and –190 and –30 were used to classify skeletal muscle and adipose tissue, respectively. Muscle sites were identified using SliceOmatic software (version 5.0; Tomovision, Montreal, QC, Canada), and the T4-SMA of the corresponding tissues within the outlined range were automatically calculated, such as the cross-sectional areas of the pectoralis, intercostals, paraspinals, serratus, and vastus muscles, along with the mean skeletal muscle density at the T4 level (T4-SMD), as shown in Fig. 2. The SMI was subsequently calculated using the relative height- or BMI-adjusted muscle mass to infer the muscle mass size; that is cross-sectional area of skeletal muscle mass at the T4 level divided by the height^2^ (or the BMI^2^) [10]. Due to the lack of an established reference value for defining sarcopenia using the T4-SMI in Asian populations, a sex-specific cut-off point analysis was performed, and “low SMI” classification was defined as an T4-SMI below the respective sex-specific quartile [11, 12, 13]. When patients were stratified by T4-SMA divided by height square, the cutoff value corresponds to the lowest quartile (Q1) of T4-SMI (Height) (male ≤69.70 cm^2^/m^2^; female ≤55.91 cm^2^/m^2^, respectively). When correcting SMA with BMI, the cutoff value corresponds to the lowest quartile (Q1) of T4-SMI (BMI) (male ≤8.10 cm^2^/kg/m^2^; female ≤5.78 cm^2^/kg/m^2^, respectively).

### 2.4 Statistical Analysis

To ensure the variables distribution patterns were identified, we applied the Shapiro-Wilk test. In cases where variables exhibited a normal distribution with continuous data, the mean and standard deviation (SD) were calculated and reported. Conversely, for variables with a continuous distribution that was not normal, the median and interquartile range (IQR) were the reported statistics. For categorical data, the reported statistics were the counts and the corresponding percentages.

During the analysis of the initial subject characteristics, several statistical tests were utilized to evaluate and compare the variables across different groups: the *t*-test was used for variables with a normal distribution, the chi-square test or Fisher’s exact test was applied for categorical variables, and the Kruskal-Wallis test was employed for variables with a skewed distribution.

Given the variation in baseline muscular conditions between genders, participants were categorized into four groups—Q1, Q2, Q3, and Q4—based on their T4-SMI values, separately for men and women. Logistic regression analyses, both univariate and multivariate, were performed to explore the correlation between T4-SMI parameters and the recurrence of AF, with adjustments for potential confounding factors. The initial model did not include any covariates, while Model 1 included adjustments for age, sex, and BMI. Model 2 built upon Model 1 by incorporating additional comorbidities such as hypertension and diabetes. Model 3 further adjusted for the duration of AF and the left atrial diameter, expanding on Model 2.

Patients were stratified into quartile groups based on gender-specific intervals to examine the link between T4-SMI and the recurrence of AF within these groups. Subgroup and interaction analyses were also conducted, considering variables such as gender, age, BMI, hypertension, and diabetes, to assess the stability of the study’s findings. All statistical analyses were conducted using Statistical Package for the Social Sciences (SPSS) software, version 25.0 (IBM Corp., Armonk, NY, USA). The threshold for statistical significance was set at a two-tailed *p* value less than 0.05.

## 3. Results

### 3.1 Patient Characteristics

Baseline characteristics of the study subjects are provided in Table 1. Eighty-two patients (12.8%) experienced AF recurrence, age and the BMI was higher in the recurrence group (24.5 ± 3.3 kg/m^2^) than that in the no recurrence group (24.4 ± 3.3 kg/m^2^), as were the disease duration (36.0 versus 12.0 months, respectively; *p* = 0.012), the concentration of BNP and left atrial diameter (44.2 ± 7.4 versus 41.7 ± 7.2 mm, respectively; *p* = 0.004). In addition, the prevalence of comorbidities such as hypertension, diabetes mellitus, stroke and coronary heart disease was also higher in the recurrence group compared with the non-recurrence group. However, the T4-SMA was significantly lower in patients in the recurrence group compared with the non-recurrence group (187.2 ± 42.6 versus 200.3 ± 46.5 cm^2^; *p* = 0.016). The T4-SMI was significantly lower in the recurrence group than that in the non-recurrence group, regardless of which of the two methods was used to adjust for the T4-SMA before calculating the T4-SMI (T4-SMI (BMI-adjusted): 7.7 ± 1.8 versus 8.3 ± 2.0 cm^2^/kg/m^2^, respectively, *p* = 0.013; T4-SMI (Height-adjusted): 68.9 ± 13.2 versus 73.0 ± 14.1 cm^2^/m^2^, respectively, *p* = 0.014). The early recurrence rate was also significantly higher in the recurrence group compared with the non-recurrence group (26.8% versus 7.9%, respectively; *p* < 0.001).

**Table 1.** **Comparison of baseline characteristics and radiographic muscle measures between recurrence group and non-recurrence group**.

		All (n = 641)	Non-recurrence group (n = 559)	Recurrence group (n = 82)	*p* value
Demographics					
	Age (y)	67.7 ± 9.3	68.0 ± 9.2	66.1 ± 10.3	0.085
	Male (%)	401	350 (62.6%)	51 (62.2%)	0.942
	Weight (kg)	66.8 ± 11.7	66.8 ± 11.7	66.6 ± 11.9	0.914
	BMI (kg/m^2^)	24.4 ± 3.3	24.4 ± 3.3	24.5 ± 3.3	0.751
	Height (m)	1.7 ± 0.1	1.7 ± 0.1	1.6 ± 0.1	0.477
	Duration of AF (months)	12.0 (2.0, 60.0)	12.0 (1.0, 60.0)	36.0 (3.0, 72.0)	0.012
	CHA_2_DS_2_-VASc	3.0 (2.0, 4.0)	3.0 (2.0, 4.0)	3.0 (1.0, 4.0)	0.416
	LAD (mm)	42.0 ± 7.3	41.7 ± 7.2	44.2 ± 7.4	0.004
	LVEF (%)	60.8 ± 9.2	60.8 ± 9.3	60.8 ± 8.7	0.977
	BNP (pg/mL)	112.2 (49.4, 212.4)	108.5 (47.2, 211.3)	136.8 (61.0, 235.4)	0.198
	LDL-C (mg/dL)	2.3 ± 0.8	2.3 ± 0.8	2.2 ± 0.7	0.310
	HDL-C (mg/dL)	1.1 ± 0.3	1.1 ± 0.3	1.1 ± 0.3	0.559
	Creatinine (µmol/L)	85.4 ± 28.7	85.8 ± 30.3	82.3 ± 14.0	0.078
Comorbidities					
	Hypertension (n, %)	405	344 (61.5%)	61 (74.4%)	0.024
	Diabetes (n, %)	123	100 (17.9%)	23 (28.0%)	0.029
	Stroke (n, %)	83	72 (12.9%)	11 (13.4%)	0.893
	Coronary heart disease (n, %)	138	120 (21.5%)	18 (22.0%)	0.921
Muscle mass status					
	T4-SMA (cm^2^)	198.6 ± 46.2	200.3 ± 46.5	187.2 ± 42.6	0.016
	SMI (BMI) (cm^2^/kg/m^2^)	8.2 ± 2.0	8.3 ± 2.0	7.7 ± 1.8	0.013
	SMI (Height) (cm^2^/m^2^)	72.5 ± 14.0	73.0 ± 14.1	68.9 ± 13.2	0.014
	Muscular density T4 (HU)	37.7 ± 5.1	37.7 ± 5.1	37.8 ± 5.5	0.845
Early recurrence (n, %)		66	44 (7.9%)	22 (26.8%)	<0.001

Values are mean ± SD or n (%) and median and interquartile range (IQR). Abbreviations: BMI, body mass index; AF, atrial fibrillation; LAD, left atrial diameter; LVEF, left ventricular ejection fraction; BNP, B-type natriuretic peptide; T4, the fourth thoracic level; SMA, skeletal muscle area; SMI, skeletal muscle index; LDL-C, low-density lipoprotein cholestero; HDL-C, high-density lipoprotein cholesterol; y, year; CHA_2_DS_2_-VASc, congestive heart failure, hypertension, age ≥75y (doubled), diabetes mellitus, stroke (doubled)-vascular disease, age 65-74 and sex category (female) scoring system.

### 3.2 Univariate and Multivariate Analysis of Recurrence of Atrial Fibrillation 

In terms of sex, 62.6% were male patients. The male patients had higher BMIs and Heights than the female patients, as well as higher values for the T4-SMA. Due to these significant differences, the male and female patients were stratified, their data were analysed separately. There was no clear cut-off point for low SMI based on chest CT at the T4 level, we divided the patients into four groups according to T4-SMI quartiles in male and female, respectively. And the lowest quartile of T4-SMI group was defined as “low SMI” group (Q1), the rest were normal group (Q2 + Q3 + Q4). And when patients were stratified according to the T4-SMA divided by height squared, “low SMI” was defined as a T4-SMI (Height) ≤69.70 cm^2^/m^2^ for male and ≤55.91 cm^2^/m^2^ for female, respectively. When patients were stratified according to the T4-SMA divided by the BMI, “low SMI” was defined as a T4-SMI (BMI) ≤8.10 cm^2^/kg/m^2^ for males and ≤5.78 cm^2^/kg/m^2^ for female, respectively.

The potential risk factors for AF recurrence initially identified from the univariate logistic regression analysis included the duration of AF (odds ratio, OR = 1.01, 95% confidence interval (CI) = 1.00–1.01, *p* = 0.001) and left atrial diameter (OR = 1.04, 95% CI = 1.01–1.08, *p* = 0.004). They also comprised the presence of hypertension (OR = 1.82, 95% CI = 1.07–3.07, *p* = 0.026), and diabetes mellitus (OR = 1.79, 95% CI = 1.06–3.03, *p* = 0.031), T4-SMA^a^ (OR = 0.94, 95% CI = 0.89–0.99, *p* = 0.017), T4-SMI^c^ (Height) (OR = 0.80, 95% CI = 0.68–0.96, *p* = 0.014) and T4-SMI^e^ (BMI) (OR = 0.20, 95% CI = 0.06–0.71, *p* = 0.013). (See Table 2 for a detailed explanation of superscript letters).

**Table 2.** **Univariate and multivariate analysis of recurrence of atrial fibrillation**.

Variable		Univariable analysis			Multivariable analysis		
		OR	(95% CI)	*p* value	OR	(95% CI)	*p* value
Age (y)		0.98	0.96–1.00	0.086			
Male		0.98	0.61–1.58	0.942			
Weight (kg)		1.00	0.98–1.02	0.914			
Height (m)		0.36	0.02–5.85	0.476			
BMI (kg/m^2^)		1.01	0.94–1.09	0.751			
Duration of AF (months)		1.01	1.00–1.01	0.001	1.01	1.00–1.01	0.008
Hypertension (n, %)		1.82	1.07–3.07	0.026	1.47	0.85–2.53	0.167
Diabetes (n, %)		1.79	1.06–3.03	0.031	1.81	1.04–3.13	0.035
Stroke (n, %)		1.05	0.53–2.07	0.893			
Coronary heart disease (n, %)		1.03	0.59–1.8	0.921			
LAD (mm)		1.04	1.01–1.08	0.004	1.05	1.02–1.08	0.003
LVEF (%)		1.00	0.97–1.03	0.977			
CHA_2_DS_2_-VASc		0.94	0.81–1.08	0.389			
LDL-C (mg/dL)		0.87	0.65–1.17	0.360			
HDL-C (mg/dL)		1.28	0.56–2.91	0.559			
Creatinine (µmol/L)		0.99	0.98–1.01	0.288			
BNP (pg/mL)		1.00	1.00–1.01	0.486			
T4-SMA^a^ (cm^2^)		0.94	0.89–0.99	0.017			
T4-SMA^b^ (cm^2^) sex-stratified quartiles							
	Q1 (male ≤196.20; female ≤139.08)	1.77	0.93–3.38	0.084			
	Q2 (male 197.08–220.61; female 139.58–156.90)	1.20	0.6–2.39	0.600			
	Q3 (male 220.83–245.76; female 157.42–175.70)	1.00	0.49–2.04	0.999			
	Q4 (male ≥245.77; female ≥175.71)	Ref					
SMI^c^ (Height) (cm^2^/m^2^)		0.80	0.68–0.96	0.014	0.81	0.68–0.98	0.026
SMI^d^ (Height) sex-stratified quartiles							
	Q1 (male ≤69.70; female ≤55.91)	1.38	0.72–2.66	0.333			
	Q2 (male 69.81–76.2; female 55.92–63.56)	1.26	0.65–2.45	0.5			
	Q3 (male 76.27–85.36; female 63.73–71.57)	1.00	0.50–2.00	0.999			
	Q4 (male ≥85.40; female ≥71.62)	Ref					
SMI^e^ (BMI) (cm²/kg/m²)		0.20	0.06–0.71	0.013			
SMI^f^ (BMI) sex-stratified quartiles		1.000					
	Q1 (male ≤8.10; female ≤5.78)	2.31	1.17–4.55	0.016			
	Q2 (male 8.11–9.14; female 5.79–6.46)	0.86	0.38–1.9	0.707			
	Q3 (male 9.15–10.09; female 6.47–7.40)	2.13	1.07–4.24	0.031			
	Q4 (male ≥10.14; female ≥7.41)	Ref					
Muscular density T4 (HU)—Total group		1.00	0.96–1.05	0.845			

T4-SMA^a^ was entered as a continuous variable per 10 cm^2^. T4-SMA^b^ sex-stratified quartiles based on separate quartiles intervals for males and females in cm^2^. SMI^c^ (Height) was entered as a continuous variable per 10 cm^2^/m^2^. SMI^d^ (Height) sex-stratified quartiles based on separate quartiles intervals for males and females in cm^2^/m^2^. SMI^e^ (BMI) was entered as a continuous variable per 10 cm^2^/kg/m^2^. SMI^f^ (BMI) sex-stratified quartiles based on separate quartiles intervals for males and females in cm^2^/kg/m^2^. Abbreviations: OR, odds ratio; CI, confidence interval.

The subsequent multifactorial logistic regression analysis revealed that the duration of AF, left atrial diameter, presence of diabetes mellitus, and T4-SMI (both BMI- and height-adjusted) were still associated with AF recurrence. Left ventricular ejection fraction was lower in the recurrence group than in the non-recurrence group. But left ventricular ejection fraction is not significant in Univariate and multivariate analyses. The reason may be there is indeed an association between AF recurrence and ejection fraction, but this association is not simply linear.

As shown in Table 2, there was a statistically negative association between the T4-SMA^b^ and risk of AF recurrence without adjustment. The OR gradually increased as the level of the T4-SMI^d^ (Height) decreased in comparison with Q4 group. The ORs across quartiles (first to third quartiles) for AF recurrence were 1.38 (95% CI = 0.72–2.66, *p* = 0.333), 1.26 (95% CI = 0.65–2.45, *p* = 0.5), and 1.00 (95% CI = 0.50–2.00, *p* = 0.999). Among those classified according to the T4-SMI^f^ (BMI), the risk of recurrence was increased to 2.31-fold in patients with AF in the lowest versus the highest T4-SMI^f^ (BMI) quartile (95% CI = 1.17–4.55, *p* = 0.016). However, no trend of sequential increase in recurrence risk with decreasing T4-SMI^f^ (BMI) was observed in the second and third quartile (Q2, OR = 0.86, 95% CI = 0.38–1.9, *p* = 0.707 versus Q3, OR = 2.13, 95% CI = 1.07–4.24, *p* = 0.031). (See Table 2 for a detailed explanation of superscript letters).

### 3.3 Associations between Baseline SMI and Recurrence of Atrial Fibrillation 

In Table 3, we further explored the association between SMI and AF recurrence by model adjustment. There were strong significant associations between decreased SMI^a^ (Height) and increased AF recurrence risk in all models. In Model 1, after adjusting for demographic factors, quartile classification results showed a trend in association between SMI^b^ (Height) and risk of AF recurrence. The ORs for recurrence in quartiles (first to third) were 1.73 (0.85–3.48), 1.45 (0.73–2.9) and 1.08 (0.53–2.18). In addition to Model 1 adjusted factors, Model 2 further adjusted the presence of diabetes and hypertension. Using the SMI^b^ (Height)-Q4 group as a reference, the risk ORs and 95% CIs for recurrence of AF in the Q1, Q2, and Q3 groups were 1.67 (0.82–3.38), 1.37 (0.69–2.75) and 1.08 (0.53–2.18), respectively. In the fully adjusted Model (Model 3), we still observe a significant trend toward an increased risk of recurrence of AF as the SMI^b^ (Height) quartile decreases. As a result of the final Model, the ORs with 95% CIs for AF recurrence comparing the first, second, and third quartile of the SMI^b^ (Height) with the fourth quartile were 1.57 (0.76–3.22), 1.42 (0.7–2.88), and 1.19 (0.58–2.44), respectively. The link between lower SMI^c^ (BMI) and the increased risk of AF recurrence remained consistent and statistically significant, regardless of the adjustments made in the model. The first and third quartiles of SMI^d^ (BMI) (Q1 and Q3) had significantly increased risk ORs for AF recurrence compared with Q4 group. Specifically, ORs for patients in the first quartile tend to be higher, with model 1 (OR = 3.32, 95% CI = 1.52–7.23), model 2 (OR = 3.15, 95% CI = 1.43–6.97), model 3 (OR = 3.16, 95% CI = 1.40–7.15). However, we failed to observe a trend of sequentially increasing risk of AF recurrence with decreasing SMI^d^ (BMI) quartiles. (See Table 3 for a detailed explanation of superscript letters).

**Table 3.** **Association between skeletal muscle index (SMI) and recurrence of atrial fibrillation**.

Variable		Model 1 (OR, 95% CI)	*p*	Model 2 (OR, 95% CI)	*p*	Model 3 (OR, 95% CI)	*p*
SMI^a^ (Height) (cm^2^/m^2^)		0.7 (0.57–0.87)	0.001	0.71 (0.57–0.88)	0.002	0.74 (0.59–0.92)	0.007
SMI^b^ (Height) sex-stratified quartiles							
	Q1 (male ≤69.70; female ≤55.91)	1.73 (0.85–3.48)	0.128	1.67 (0.82–3.38)	0.155	1.57 (0.76–3.22)	0.222
	Q2 (male 69.81–76.2; female 55.92–63.56)	1.45 (0.73–2.9)	0.289	1.37 (0.69–2.75)	0.369	1.42 (0.7–2.88)	0.325
	Q3 (male 76.27–85.36; female 63.73–71.57)	1.08 (0.53–2.18)	0.836	1.08 (0.53–2.18)	0.838	1.19 (0.58–2.44)	0.643
	Q4 (male ≥85.40; female ≥71.62)	1 (Reference)		1 (Reference)		1 (Reference)	
SMI^c^ (BMI) (cm^2^/kg/m^2^)		0.20 (0.00–0.14)	<0.00	0.03 (0.00–0.18)	<0.00	0.31 (0.00–0.24)	0.001
SMI^d^ (BMI) sex-stratified quartiles							
	Q1 (male ≤8.10; female ≤5.78)	3.32 (1.52–7.23)	0.003	3.15 (1.43–6.97)	0.005	3.16 (1.40–7.15)	0.006
	Q2 (male 8.11–9.14; female 5.79–6.46)	1.05 (0.46–2.41)	0.912	1.00 (0.43–2.33)	0.997	1.04 (0.44–2.44)	0.938
	Q3 (male 9.15–10.09; female 6.47–7.40)	2.37 (1.17–4.80)	0.016	2.38 (1.16–4.87)	0.018	2.58 (1.24–5.36)	0.011
	Q4 (male ≥10.14; female ≥7.41)	1 (Reference)		1 (Reference)		1 (Reference)	

SMI^a^ (Height) was entered as a continuous variable per 10 cm^2^/m^2^. SMI^b^ (Height) sex-stratified quartiles based on separate quartiles intervals for males and females in cm^2^/m^2^. SMI^c^ (BMI) was entered as a continuous variable per 10 cm^2^/kg/m^2^. SMI^d^ (BMI) sex-stratified quartiles based on separate quartiles intervals for males and females in cm^2^/kg/m^2^. Model 1 adjusted for age, sex, BMI in continuous analyses, no adjustment for sex in sex-stratified quartiles. Model 2 adjusted as for model 1, additionally adjusted for hypertension, diabetes. Model 3 adjusted as for model 2, additionally adjusted for duration of atrial fibrillation (AF), LAD.

### 3.4 Subgroup Analyses

**Fig. 3.**
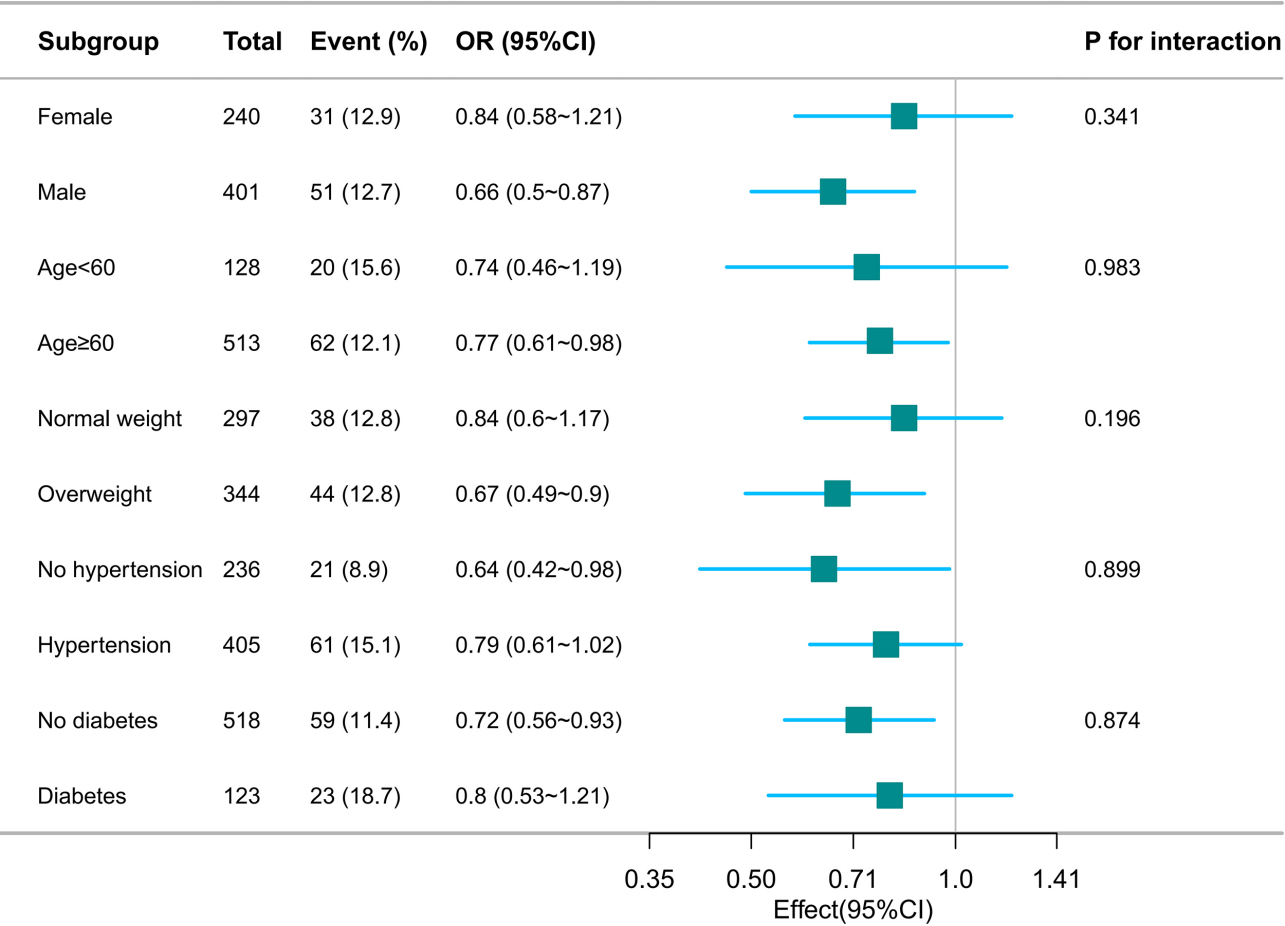
**Subgroup analysis between SMI (Height) and recurrence of atrial fibrillation**.

This study stratified all research subjects by gender, age and BMI, the presence of hypertension and diabetes, and adjusted variables other than stratified variables, including left atrial diameter and duration of AF. In subgroup analyses (Fig. 3), the results remained approximately consistent when grouped by sex (*p* value for interaction = 0.314). In addition, the results of the study showed that the risk of AF recurrence, regardless of age above or below 60 years (*p* value for interaction = 0.983) and overweight/obesity (*p* value for interaction = 0.196), whether the patient suffers from hypertension (*p* value for interaction = 0.899) or diabetes (*p* value for interaction = 0.874) was significantly associated with SMI (Height).

## 4. Discussion

The main findings of this study were: First, patients with a low relative muscle mass were more likely to experience AF recurrence. Second, a low T4-SMI adjusted for either BMI or height exhibited predictive value for assessing the likelihood of adverse outcomes in patients with AF who had undergone radiofrequency ablation, with the height adjustment being superior to the BMI adjustment in terms of the diagnostic accuracy for “low SMI”. Thirdly, subgroup analysis was also conducted to stratify patients based on gender, age, and BMI, the presence of hypertension and diabetes, in order to fully demonstrate the role of T4-SMI (Height) in predicting AF recurrence after ablation. These findings have important clinical implications, as methods for improving muscle status before catheter ablation in patients with AF could help reduce recurrence rates.

Clinically, AF occurrence and the expected prognosis are closely related to nutritional status [14]. As an indicator for assessing obesity and nutritional status in patients with AF, every five-unit increase in the BMI has been shown to be associated with a 13% increase in AF recurrence post-ablation [2, 15]. Additional evidence suggests that patients with obesity with AF have a lower risk of all-cause mortality compared with that in patients with AF who have a normal BMI, demonstrating an apparent obesity paradox [16]. Highlighting that the BMI does not accurately reflect one’s body composition is important, as it cannot distinguish between the relative weight of various components such as that of fat, muscle, and bone. Therefore, body composition measurements may be a better indicator of the risk of AF recurrence than BMI alone for assessing individual metabolic consequences [5].

Various imaging techniques have been utilized to estimate muscle mass or lean body mass, including magnetic resonance imaging (MRI), CT, DXA, and BIA. DXA and BIA cannot provide direct measurements and may over- or underestimate an individual’s actual muscle mass, especially in those who are obese or have experienced heart failure. However, CT analysis allows for an accurate and specific examination of the SMA and muscle density from individual cross-sections and is considered the “gold standard” imaging modality for estimating muscle mass.

Traditionally, CT images at the L3 level have been used to quantify skeletal muscle and fat mass. However, the L3 cut-off for determining sarcopenia is only applicable to patients undergoing abdominal CT imaging. In patients undergoing radiofrequency ablation of AF, abdominal CT images are not readily available. A large population-based study established more specific cut-offs for normal values of the cross-sectional area of muscle tissues based on CT imaging performed at different levels [17]. The pectoral muscle area measured using chest CT correlates with whole-body skeletal muscle mass measured by the BIA method [18]. The planes selected for most of the recent studies have been at the T4 or T12 level [9, 19]. Other studies have used a low skeletal muscle mass, as determined by chest CT, as a poor prognostic indicator in patients with acute pulmonary embolism [19], chronic obstructive pulmonary disease [18], left ventricular assist device implantation [20], coronavirus disease 2019 infection [21], and lung cancer [22]. Zuckerman *et al*. [23] also reported that the T4-SMA correlated with markers of frailty in older adults undergoing cardiac surgery. Thus, the T4-SMA measured in the present study may reflect the whole-body muscle mass and provide a quick and easy means of identifying a reduction in skeletal muscle.

A study that has assessed the risk of AF based on body size and composition measurements in older adults have suggested that body size, rather than the BMI, may be a more significant indicator of the risk of AF development [24]. There is a linear correlation between height and the incidence rate of atrial fibrillation in the elderly, reflecting the lower lean body mass in elders and its closer dependence on height. However, our study focused on individuals of all ages, and it is important to note that there is a greater lean mass per unit of body weight in this age group compared to older participants. So the same relationships between height and AF that have been documented in older individuals may not apply equally in younger people. Thus the patient’s whole body size should be considered when assessing the presence of sarcopenia (Here it refers to “low SMI”). In this study, the relative muscle mass was adjusted for either height or BMI. The diagnostic prevalence of “low SMI” varies depending on the correction methods used [7].

Low relative muscle mass has been shown to be associated with poor health outcomes in patients with CVD and it may serve as a surrogate marker for vulnerability to acute stressors such as cardiac surgery [25]. The association of low relative muscle mass with CVD is driven by various underlying mechanisms, some of which include mitochondrial dysfunction in muscle tissues, oxidative stress, excessive inflammatory states, microvascular endothelial dysfunction, and several metabolic disorders, such as metabolic syndrome, insulin resistance, and non-alcoholic fatty liver disease [26, 27, 28]. Furthermore, both low relative muscle mass and CVD are affected by similar lifestyle factors, such as malnutrition and physical inactivity [29]. To date, body composition has not been included as a standard indicator of frailty in patients undergoing major cardiovascular surgery.

Sarcopenia in older patients is associated with electrocardiographic abnormalities, including AF [30]. Lower muscle mass, and higher fat mass, indirectly calculated using body composition prediction equations, are known to be associated with an increased AF risk [31]. An *in vivo* study in a mouse model in which the myocardium specifically expressed a muscle growth inhibitory prepeptide to inhibit the effects of muscle growth inhibitory hormone demonstrated the presence of atrial enlargement and fibrosis and AF promotion [32]. A clinical study also reported an increased risk of AF in middle-aged and older adults without clinical heart failure in whom sarcopenia was identified using DXA [6]. The association between sarcopenia and AF is only significant in overweight/obese participants. However, to our knowledge, the present study is the first to examine the relationship between CT-diagnosed low relative muscle mass and AF recurrence following radiofrequency ablation. Due to the lack of reference values for low relative muscle mass at the T4 level, the lowest quartile of T4-SMI group was defined as “low SMI” group. Furthermore, this study found that, a lower height-adjusted T4-SMI significantly correlated with AF recurrence post-ablation, regardless of overweight/obesity. Low muscle mass participates in a mutually reinforcing and influential relationship with hypertension and diabetes. In turn, hypertension and diabetes mellitus, which are confirmed cardiovascular risk factors, simultaneously contribute to AF recurrence. In the present study, the significant relationship between a low SMI and AF recurrence after radiofrequency ablation remained, even after adjustment for above mentioned comorbidities and other risk factors.

As mentioned earlier, the dysfunction of skeletal muscle mitochondrial activity and the increase in insulin resistance can mutually amplify with aging. However, AF may result from energy metabolism disorders caused by mitochondrial dysfunction, inflammation, and oxidative stress, which as key upstream mediators of atrial electrical and structural remodelling are also involved in the pathophysiological processes that drive AF by influencing atrial ion channel alterations [33, 34, 35]. Patients with AF exhibit greater hyperinsulinemia resistance, which exacerbates the delay in atrial conduction velocity, leading to persistent electrophysiological remodelling in atrial tissue and increasing the likelihood of recurrence following ablation [36]. The effect of low muscle mass on AF occurrence can also be impacted by sex hormones; for example, in men, the levels of testosterone, which acts as an anabolic hormone in muscle, decrease with age, and testosterone deficiency has been shown to be associated with an increased risk of AF [37]. In contrast, in women, hormones such as oestradiol and progesterone can ameliorate insulin resistance and mitochondrial dysfunction by altering mitochondrial H2O2 production in skeletal muscle, and the loss of oestrogen in menopausal women results in a significant decline in muscle performance, leading to autonomic disturbances that increase the susceptibility to AF [38]. Learning more about the aforementioned mechanisms is expected to advance the current understanding of the pathophysiological processes that mediate the correlation between low relative muscle mass and AF recurrence after radiofrequency ablation.

Previous research study has stated that at least some cases of low muscle mass, particularly those with obesity, are preventable [39]. Therefore, therapeutic interventions that actively to increase aerobic capacity and muscle mass are likely to be effective in preventing AF recurrence, including resistance training exercises and nutritional strategies that increase protein and micronutrient intake, such as supplementation of omega-3 polyunsaturated fatty acid and vitamin D [40, 41]. The 2020 European Society of Cardiology (ESC) guidelines for the diagnosis and management of AF state that there is a U-shaped relationship between exercise intensity and the incidence of AF, with regular moderate-intensity exercise performed over a long period of time being effective in reducing the risk of AF. However, those guidelines do not recommend high-intensity exercise [42]. The ACTIVE-AF study of the ESC published in 2021 showed that performing aerobic exercise over a six-month period helped minimise AF recurrence [43]. However, the 2023 American College of Cardiology/American Heart Association/American College of Chest Physicians/Heart Rhythm Society (ACC/AHA/ACCP/HRS) Guidelines for the Diagnosis and Management of Atrial Fibrillation recommend 210 min per week of moderate-to-vigorous exercise training to improve cardiac rehabilitation in patients with AF who undergo ablation [44]. However, vigorous exercise increases the risk of AF, possibly by promoting myoelectric and anatomical remodelling of atrial tissues [45]. In addition, both testosterone replacement therapy and treatment with growth hormone analogues promote muscle growth and fat loss. Although active counselling of patients with obesity or overweight with AF is an effective intervention for facilitating appropriate weight loss, the greatest benefits of weight loss depend on the ability to preserve muscle tissues. Therefore, optimising patient’s body composition by increasing lean body mass and decreasing fat weight is important, a strategy that is more effective than simply targeting BMI reduction [46].

This study has several limitations. First, the sample size was limited, and all patients were from a single treatment centre. Second, this study was retrospective and participant muscle strength data was not available. Third, skeletal muscle composition and mass may not have been precisely quantified, as the main focus of this study was on SMA and low SMI. In addition, muscle status varies between races, sexes, and age groups, and the prevalence of low relative muscle mass and its association with clinical outcomes can vary widely depending on the assessment methods. Although the height-based adjustment resulted in better predictive ability of the model compared with that of the BMI-based adjustment in the present study, the superiority of some methods of SMI correction have not been conclusively demonstrated. Therefore, establishing a universal definition of sarcopenia remains difficult. Fourthly, we attempt to include various diseases in our exclusion criteria; However, some diseases that may affect muscle mass, such as chronic obstructive pulmonary disease, heart failure, chronic kidney disease, and cachexia, have not been excluded.

Instead of using methods such as BIA and DXA to assess lean body mass, this study used CT-based imaging of cross-sectional areas to rapidly quantify skeletal muscle mass to calculate the BMI- or height-adjusted T4-SMI as a parameter for assessing muscle mass, not only in the elderly but also in individuals in other age ranges and of both sexes to determine its ability to predict AF recurrence after radiofrequency ablation. Low relative muscle mass is likely to remain undetected in the clinical setting. The present study demonstrated that pre-procedural CT scanning can help detect “low SMI”, allowing for earlier intervention with nutritional or exercise therapies to reduce the loss of skeletal muscle and the accumulation of adipose tissue in patients, thereby improving their clinical prognosis. The data emphasise the importance of preventing AF, regardless of gender, age or overweight/obesity, by protecting against muscle deterioration.

## 5. Conclusions

This is the first study to evaluate the predictive value of the SMI measured at the T4 level using CT in patients with AF. Both BMI- or height-adjusted T4-SMI had good sensitivity for predicting AF recurrence. The height adjustment performed better than the BMI adjustment in that regard. A decreased T4-SMI adjusted for height was strongly associated with an increased risk of AF recurrence following radiofrequency ablation, irrespective of patients’ gender, age, or status of being overweight/obese. The correlation between T4-SMI (height) and AF recurrence was fully validated by constructing multiple models, and adjustment for different sets of covariates barely altered the results.

## Availability of Data and Materials

All data generated or analyzed during this study are included in this article. Further enquiries can be directed to the corresponding author on reasonable request.

## References

[1] Shi S, Tang Y, Zhao Q, Yan H, Yu B, Zheng Q (2022). Prevalence and risk of atrial fibrillation in China: A national cross-sectional epidemiological study. *The Lancet Regional Health. Western Pacific*.

[2] Powell-Wiley TM, Poirier P, Burke LE, Després JP, Gordon-Larsen P, Lavie CJ (2021). Obesity and Cardiovascular Disease: A Scientific Statement From the American Heart Association. *Circulation*.

[3] Tikkanen E, Gustafsson S, Knowles JW, Perez M, Burgess S, Ingelsson E (2019). Body composition and atrial fibrillation: a Mendelian randomization study. *European Heart Journal*.

[4] Fenger-Grøn M, Overvad K, Tjønneland A, Frost L (2017). Lean Body Mass Is the Predominant Anthropometric Risk Factor for Atrial Fibrillation. *Journal of the American College of Cardiology*.

[5] Gao K, Cao LF, Ma WZ, Gao YJ, Luo MS, Zhu J (2022). Association between sarcopenia and cardiovascular disease among middle-aged and older adults: Findings from the China health and retirement longitudinal study. *EClinicalMedicine*.

[6] Xia MF, Chen LY, Wu L, Ma H, Li XM, Li Q (2021). Sarcopenia, sarcopenic overweight/obesity and risk of cardiovascular disease and cardiac arrhythmia: A cross-sectional study. *Clinical Nutrition (Edinburgh, Scotland)*.

[7] Kim KM, Jang HC, Lim S (2016). Differences among skeletal muscle mass indices derived from height-, weight-, and body mass index-adjusted models in assessing sarcopenia. *The Korean Journal of Internal Medicine*.

[8] Cruz-Jentoft AJ, Baeyens JP, Bauer JM, Boirie Y, Cederholm T, Landi F (2010). Sarcopenia: European consensus on definition and diagnosis: Report of the European Working Group on Sarcopenia in Older People. *Age and Ageing*.

[9] Arayne AA, Gartrell R, Qiao J, Baird PN, Yeung JM (2023). Comparison of CT derived body composition at the thoracic T4 and T12 with lumbar L3 vertebral levels and their utility in patients with rectal cancer. *BMC Cancer*.

[10] Heymsfield SB, Heo M, Thomas D, Pietrobelli A (2011). Scaling of body composition to height: relevance to height-normalized indexes. *The American Journal of Clinical Nutrition*.

[11] Moon SW, Choi JS, Lee SH, Jung KS, Jung JY, Kang YA (2019). Thoracic skeletal muscle quantification: low muscle mass is related with worse prognosis in idiopathic pulmonary fibrosis patients. *Respiratory Research*.

[12] Cawthon PM, Peters KW, Shardell MD, McLean RR, Dam TTL, Kenny AM (2014). Cutpoints for low appendicular lean mass that identify older adults with clinically significant weakness. *The Journals of Gerontology. Series A, Biological Sciences and Medical Sciences*.

[13] Moon SW, Lee SH, Woo A, Leem AY, Lee SH, Chung KS (2022). Reference values of skeletal muscle area for diagnosis of sarcopenia using chest computed tomography in Asian general population. *Journal of Cachexia, Sarcopenia and Muscle*.

[14] Anaszewicz M, Budzyński J (2017). Clinical significance of nutritional status in patients with atrial fibrillation: An overview of current evidence. *Journal of Cardiology*.

[15] Wong CX, Sullivan T, Sun MT, Mahajan R, Pathak RK, Middeldorp M (2015). Obesity and the Risk of Incident, Post-Operative, and Post-Ablation Atrial Fibrillation: A Meta-Analysis of 626,603 Individuals in 51 Studies. *JACC. Clinical Electrophysiology*.

[16] Elagizi A, Kachur S, Lavie CJ, Carbone S, Pandey A, Ortega FB (2018). An Overview and Update on Obesity and the Obesity Paradox in Cardiovascular Diseases. *Progress in Cardiovascular Diseases*.

[17] Derstine BA, Holcombe SA, Ross BE, Wang NC, Su GL, Wang SC (2018). Skeletal muscle cutoff values for sarcopenia diagnosis using T10 to L5 measurements in a healthy US population. *Scientific Reports*.

[18] O’Brien ME, Zou RH, Hyre N, Leader JK, Fuhrman CR, Sciurba FC (2023). CT pectoralis muscle area is associated with DXA lean mass and correlates with emphysema progression in a tobacco-exposed cohort. *Thorax*.

[19] Meyer HJ, Kardas H, Schramm D, Bär C, Wienke A, Borggrefe J (2023). CT-defined pectoralis muscle mass and muscle density are associated with mortality in acute pulmonary embolism. A multicenter analysis. *Clinical Nutrition (Edinburgh, Scotland)*.

[20] Teigen LM, John R, Kuchnia AJ, Nagel EM, Earthman CP, Kealhofer J (2017). Preoperative Pectoralis Muscle Quantity and Attenuation by Computed Tomography Are Novel and Powerful Predictors of Mortality After Left Ventricular Assist Device Implantation. *Circulation. Heart Failure*.

[21] Koehler J, Boirie Y, Bensid L, Pereira B, Ghelis N, Dupuis C (2022). Thoracic sarcopenia as a predictive factor of SARS-COV2 evolution. *Clinical Nutrition (Edinburgh, Scotland)*.

[22] Sun C, Anraku M, Kawahara T, Karasaki T, Kitano K, Nagayama K (2020). Prognostic significance of low pectoralis muscle mass on preoperative chest computed tomography in localized non-small cell lung cancer after curative-intent surgery. *Lung Cancer (Amsterdam, Netherlands)*.

[23] Zuckerman J, Ades M, Mullie L, Trnkus A, Morin JF, Langlois Y (2017). Psoas Muscle Area and Length of Stay in Older Adults Undergoing Cardiac Operations. *The Annals of Thoracic Surgery*.

[24] Karas MG, Yee LM, Biggs ML, Djoussé L, Mukamal KJ, Ix JH (2016). Measures of Body Size and Composition and Risk of Incident Atrial Fibrillation in Older People: The Cardiovascular Health Study. *American Journal of Epidemiology*.

[25] Gallone G, Depaoli A, D’Ascenzo F, Tore D, Allois L, Bruno F (2022). Impact of computed-tomography defined sarcopenia on outcomes of older adults undergoing transcatheter aortic valve implantation. *Journal of Cardiovascular Computed Tomography*.

[26] Lee SW, Youm Y, Lee WJ, Choi W, Chu SH, Park YR (2015). Appendicular skeletal muscle mass and insulin resistance in an elderly korean population: the korean social life, health and aging project-health examination cohort. *Diabetes & Metabolism Journal*.

[27] Zhu Q, Zhang T, Cheang I, Lu X, Shi M, Zhu X (2023). Negative association between triglyceride glucose index and BMI-adjusted skeletal muscle mass index in hypertensive adults. *BMC Musculoskeletal Disorders*.

[28] Johannsen DL, Conley KE, Bajpeyi S, Punyanitya M, Gallagher D, Zhang Z (2012). Ectopic lipid accumulation and reduced glucose tolerance in elderly adults are accompanied by altered skeletal muscle mitochondrial activity. *The Journal of Clinical Endocrinology and Metabolism*.

[29] Oterdoom LH, Gansevoort RT, Schouten JP, de Jong PE, Gans ROB, Bakker SJL (2009). Urinary creatinine excretion, an indirect measure of muscle mass, is an independent predictor of cardiovascular disease and mortality in the general population. *Atherosclerosis*.

[30] Heshmat R, Shafiee G, Ostovar A, Fahimfar N, Maleki Birjandi S, Jabbari M (2021). Relationship Between Sarcopenia and Electrocardiographic Abnormalities in Older People: The Bushehr Elderly Health Program. *Frontiers in Medicine*.

[31] Woo HG, Kang MK, Song TJ (2023). Association of predicted body composition with occurrence of atrial fibrillation. *Frontiers in Cardiovascular Medicine*.

[32] Rosenberg MA, Das S, Quintero Pinzon P, Knight AC, Sosnovik DE, Ellinor PT (2012). A Novel Transgenic Mouse Model of Cardiac Hypertrophy and Atrial Fibrillation. *Journal of Atrial Fibrillation*.

[33] Harada M, Tadevosyan A, Qi X, Xiao J, Liu T, Voigt N (2015). Atrial Fibrillation Activates AMP-Dependent Protein Kinase and its Regulation of Cellular Calcium Handling: Potential Role in Metabolic Adaptation and Prevention of Progression. *Journal of the American College of Cardiology*.

[34] van Bilsen M, Smeets PJH, Gilde AJ, van der Vusse GJ (2004). Metabolic remodelling of the failing heart: the cardiac burn-out syndrome?. *Cardiovascular Research*.

[35] Tse G, Yan BP, Chan YWF, Tian XY, Huang Y (2016). Reactive Oxygen Species, Endoplasmic Reticulum Stress and Mitochondrial Dysfunction: The Link with Cardiac Arrhythmogenesis. *Frontiers in Physiology*.

[36] Wang Z, Wang YJ, Liu ZY, Li Q, Kong YW, Chen YW (2023). Effect of Insulin Resistance on Recurrence after Radiofrequency Catheter Ablation in Patients with Atrial Fibrillation. *Cardiovascular Drugs and Therapy*.

[37] Ko D, Rahman F, Schnabel RB, Yin X, Benjamin EJ, Christophersen IE (2016). Atrial fibrillation in women: epidemiology, pathophysiology, presentation, and prognosis. *Nature Reviews. Cardiology*.

[38] Abbatecola AM, Paolisso G, Fattoretti P, Evans WJ, Fiore V, Dicioccio L (2011). Discovering pathways of sarcopenia in older adults: a role for insulin resistance on mitochondria dysfunction. *The Journal of Nutrition, Health & Aging*.

[39] Sakuma K, Yamaguchi A (2013). Sarcopenic obesity and endocrinal adaptation with age. *International Journal of Endocrinology*.

[40] Waters DL, Baumgartner RN, Garry PJ, Vellas B (2010). Advantages of dietary, exercise-related, and therapeutic interventions to prevent and treat sarcopenia in adult patients: an update. *Clinical Interventions in Aging*.

[41] Springer J, Springer JI, Anker SD (2017). Muscle wasting and sarcopenia in heart failure and beyond: update 2017. *ESC Heart Failure*.

[42] Hindricks G, Potpara T, Dagres N, Arbelo E, Bax JJ, Blomström-Lundqvist C (2021). 2020 ESC Guidelines for the diagnosis and management of atrial fibrillation developed in collaboration with the European Association for Cardio-Thoracic Surgery (EACTS): The Task Force for the diagnosis and management of atrial fibrillation of the European Society of Cardiology (ESC) Developed with the special contribution of the European Heart Rhythm Association (EHRA) of the ESC. *European Heart Journal*.

[43] Visseren FLJ, Mach F, Smulders YM, Carballo D, Koskinas KC, Bäck M (2022). 2021 ESC Guidelines on cardiovascular disease prevention in clinical practice. *European Journal of Preventive Cardiology*.

[44] Joglar JA, Chung MK, Armbruster AL, Benjamin EJ, Chyou JY, Cronin EM (2024). 2023 ACC/AHA/ACCP/HRS Guideline for the Diagnosis and Management of Atrial Fibrillation: A Report of the American College of Cardiology/American Heart Association Joint Committee on Clinical Practice Guidelines. *Circulation*.

[45] Elliott AD, Linz D, Verdicchio CV, Sanders P (2018). Exercise and Atrial Fibrillation: Prevention or Causation?. *Heart, Lung & Circulation*.

[46] Cai X, Liu M, Xu X, Zhang S, Huang R, Wang P (2024). Cardiovascular effects of weight loss in old adults with overweight/obesity according to change in skeletal muscle mass. *Journal of Cachexia, Sarcopenia and Muscle*.

